# An Amyand Hernia With Concurrent Appendicitis Secondary to a Neuroendocrine Neoplasm: A Case Report

**DOI:** 10.7759/cureus.54894

**Published:** 2024-02-25

**Authors:** Jason Zouki, Anoj Dharmawardhane

**Affiliations:** 1 General Surgery, Toowoomba Hospital, Toowoomba, AUS

**Keywords:** laparoscopic appendicectomy, indirect inguinal hernia, appendiceal neuroendocrine tumor, s: acute appendicitis, amyand’s hernia

## Abstract

Amyand’s hernia (AH) describes the rare instance of a vermiform appendix within an inguinal hernia. Primary appendiceal neoplasms are also rare with the majority of cases being found incidentally during routine histopathology. This case reports the management of a 15-year-old male, who presented to the emergency department with acute appendicitis located within an indirect right inguinal hernia, which was ultimately secondary to a neuroendocrine tumor (NET) with serosal involvement. Intraoperative findings included macroscopic appendicitis with no evidence of perforation. Histopathology returned as a neuroendocrine tumor (pT4) with involved proximal margin and curative treatment was undertaken with a caecectomy which returned no residual malignancy. Key considerations include management options of peritoneal spread within the inguinal canal and recommended management NET in the context of an AH. It is important to understand the varied presentations of common surgical diagnosis such as appendicitis and underlying malignancy should always be considered a differential.

## Introduction

Amyand’s hernia (AH) describes the presence of the vermiform appendix in an inguinal hernia and accounts for 0.1% of all cases of appendicitis and 0.4% to 0.6% of all cases of inguinal hernias [1]. AH occur due to a patent processus vaginalis and are up to three times more common in the paediatric population, with the overwhelming majority identified in males or in females who are post-menopausal [2]. The underlying pathophysiology is not clearly understood, however the presence of a congenital fibrous band between the appendix and scrotum has been proposed as an underlying cause [2]. AH have a poor rate of pre-operative identification due to the varied clinical presentation and low detection rates on ultrasound [3]. The presence of macroscopic inflammation at the time of appendicectomy increases the rate of post-operative infection to around 5% and should be a key determinant of the type of hernia repair [2]. 

Primary appendiceal neoplasms are rare entities with diverse pathophysiology and a wide spectrum of mortality based on the tumour type and staging. Appendiceal malignancies are defined by the World Health Organisation (WHO) classification into epithelial (mucinous, non-mucinous adenocarcinoma, and signet ring cell tumors) and non-epithelial lesions (neuroendocrine tumours [NETs], lymphomas, and sarcomas) [4]. Appendiceal NETs account for 65% of appendiceal tumours, but are only found in 0.5% to 1% of all appendicectomies, the majority of which are incidental [5].

## Case presentation

A 15-year-old male presented to the emergency department with 24 hours of migratory pain to the right iliac fossa, subjective fevers and anorexia consistent with a clinical diagnosis of acute appendicitis. He denied any associated bowel changes, weight loss, dysuria, testicular pain or new groin masses. The patient’s past medical history was limited to autism and his surgical history consisted of an open left inguinal herniotomy at the age of 10 for an indirect inguinal hernia. On review he was tachypneic to 24 in the context of pain, but afebrile with a heart rate of 80 beats per minute and regular. Abdominal examination revealed right iliac fossa tenderness with a positive Rovsing sign, but no hernia was detected. Initial investigations revealed a normal white cell count of 10.0 x 10^9/L with an elevated C-reactive protein of 55 mg/L. The remainder of the blood tests were unremarkable including a full blood count, liver function test, urea and electrolytes. The patient was diagnosed with appendicitis based on history and clinical findings and progressed to a laparoscopic appendicectomy with no pre-operative imaging.

**Figure 1:**
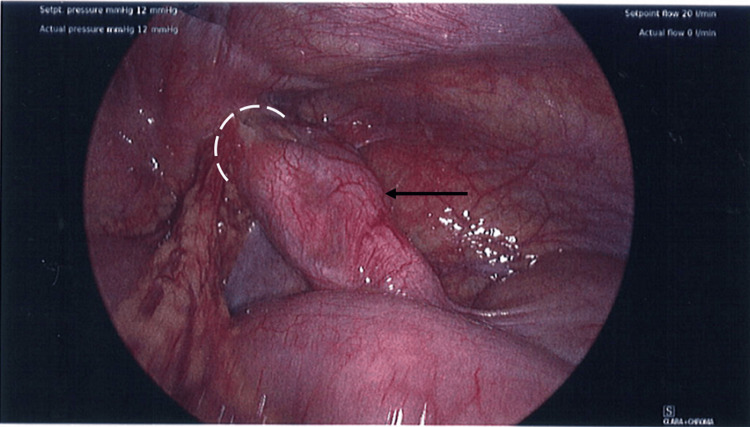
Intraoperative picture showing macroscopic appendicitis with an Amyand hernia White semicircle: Indirect (right) inguinal hernia Black arrow: Appendix with macroscopic inflammation

Intraoperatively, an AH was found with macroscopic appendicitis within an indirect inguinal hernia (Figure 1), however no intra-abdominal free fluid or evidence of perforation was found. The previous hernia repair was intact and the decision was made to not proceed with a right inguinal hernia repair secondary to the presence of macroscopic inflammation and a risk of bacterial translocation with subsequent mesh infection. 

**Figure 2:**
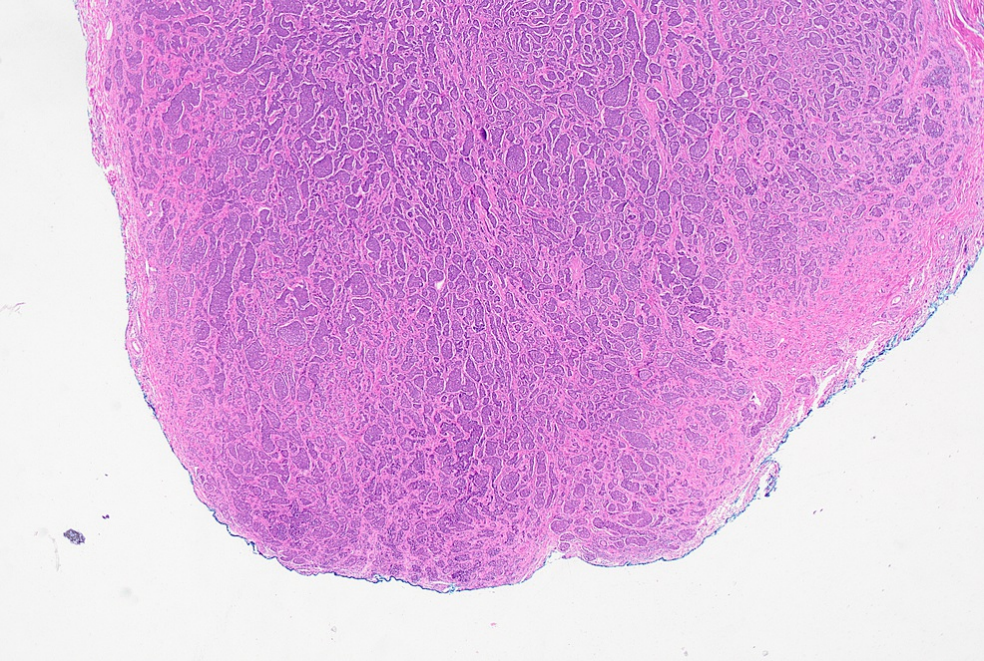
Haemotoxylin and Eosin stain of the appendix showing serosal involvement (pT4 staging)

**Figure 3:**
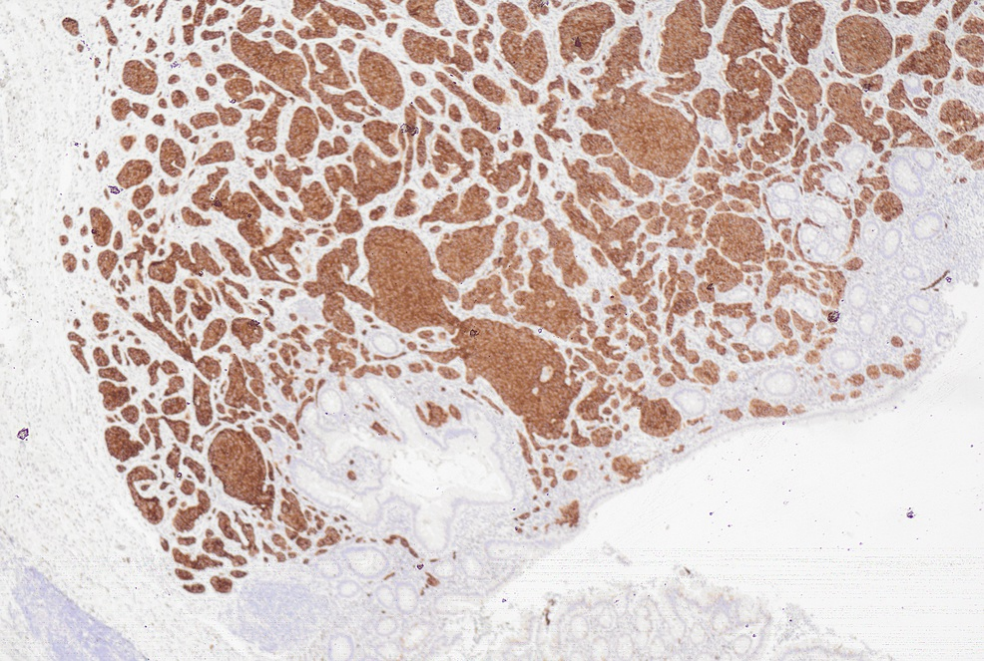
Histopathology slide with chromogranin A-stained appendix showing neuroendocrine tumour in brown

The patient was discharged the following morning with no concerns, however he presented one week later with loose stools and generalised abdominal pain. A computerized tomography (CT) scan failed to demonstrate any intra-abdominal collections or post-operative complications and he was managed with a course of oral antibiotics after a stool specimen confirmed the presence of Clostridium difficile. Appendiceal histology returned the following week, demonstrating ‘acute appendicitis with a well-differentiated neuroendocrine neoplasm in the proximal appendix and possible proximal margin involvement (Figure 2). The maximum tumour diameter is 11mm with invasion through the muscularis propria (Figure 3) into subserosa (pT4) and a Ki-67 index less than 1%’.

A chromogranin A level returned normal and a DOTATATE Positron Emission Tomography (PET) scan revealed an avid right inguinal lymph node, however there were no features of nodal malignancy on the ultrasound scan and the scan was otherwise unremarkable. A multi-disciplinary team meeting was held with the surgical and oncological team regarding benefits of a caecectomy against a limited right hemicolectomy. The patient was followed up in the outpatient setting with his family, and he decided to proceed with a caecectomy which returned with no residual malignancy. A post-operatively colonoscopy one month after his caecectomy was unremarkable with nil lesions identified, and the patient underwent a clinical review at six months which was unremarkable. He was discharged from clinic with a curative prognosis and no further follow-up required. 

## Discussion

Appendicitis is a common surgical presentation commonly managed via laparoscopic appendicectomy. The presence of an inflamed appendix within an inguinal hernia is rare and accounts for only 0.1% of appendicectomies. Neoplasms of the appendix are also rare entities and are found in less than 2% of all appendicectomies [6], with only 0.3% - 0.9% diagnosed as a NET. Losanoff and Basson have proposed a classification for surgical management of AH, however the varied approaches are heavily reliant on pre-operative identification of AH, which is considered difficult to diagnose [7].

The management of a NET is well described, and dependent on the size of the neoplasm as well as the presence of high-risk features, including mesoappendiceal involvement, location at the base of the appendix, Ki-67 >2%, angioinvasion, neuroinvasion or involved surgical margins [8]. Given the involved margin and proximal location at the base of the appendix, definitive treatment with a right hemicolectomy, or yearly cross-sectional imaging with CT or Magnetic Resonance Imaging (MRI) is suggested. Of note, the patient was initially staged as pT4NxMx (Histopathology slide 4), requiring consideration of peritoneal carcinomatosis, which has been reported as commonly as 10% to 33% of NET [9]. In the presence of a well-differentiated malignancy, with a Ki-67% <1% and a reassuring CT/PET, the decision was made to manage the inguinal canal expectantly. Interestingly, despite a high rate of nodal involvement around 47% in NET between 1-2cm, Rossi and Patel report a 0% recurrence rate if the primary lesion is <2cm [10]. Definitively, a caecectomy was planned with curative intent to resect the involved margin and avoid the likely unnecessary morbidity of a right hemicolectomy.

Furthermore, the patient underwent a colonoscopy post-operatively to exclude synchronous lesions, which are present in up to 22% of patients with a NET [10], however this was unremarkable with no lesions identified.

## Conclusions

Appendicitis is a common surgical presentation which may is typically managed via laparoscopic resection. Understanding of the varied ways in which it may present and the best proposed management outcomes it critical. Occasionally it may present as part of a secondary condition, such as AH and a differential diagnosis in the underlying cause must always include malignancy. Furthermore, an understanding of proposed surgical managements as reported by Losanoff and Basson may help with preoperatively planning during these rare cases.
